# Type 2 diabetes mellitus and the risk of abnormal spermatozoa: A Mendelian randomization study

**DOI:** 10.3389/fendo.2022.1035338

**Published:** 2022-11-02

**Authors:** Mengyuan Dai, Weijie Guo, San Zhu, Guidong Gong, Mei Chen, Zhuoling Zhong, Junling Guo, Yaoyao Zhang

**Affiliations:** ^1^ Department of Obstetrics and Gynecology of West China Second University Hospital, BMI Center for Biomass Materials and Nanointerfaces, College of Biomass Science and Engineering, Sichuan University, Chengdu, Sichuan, China; ^2^ Key Laboratory of Birth Defects and Related of Women and Children of Ministry of Education, West China Second University Hospital, Sichuan University, Chengdu, China; ^3^ State Key Laboratory of Polymer Materials Engineering, Sichuan University, Chengdu, China

**Keywords:** T2DM, abnormal spermatozoa, SNPs, Mendelian randomization, GWAS

## Abstract

Abnormal spermatozoa can not only reduce the fertilization rate, but also prolong the natural conception time and even increase the risk of spontaneous miscarriage. Diabetes mellitus (DM) has become a major global health problem, and its incidence continues to rise, while affecting an increasing number of men in their reproductive years. Type 2 Diabetes Mellitus (T2DM), accounting for about 85-95% of DM, is closely related to the development of sperm. However, the exact association between T2DM and abnormal spermatozoa remains unclear. Herein, we designed a Two-sample Mendelian randomization (MR) study to explore the causal association between T2DM and abnormal spermatozoa risk in European population data which come from the GWAS summary datasets. We selected 9 single nucleotide polymorphisms (SNPs) of T2DM (exposure data) as instrumental variables (IVs), and then retrieved the suitable abnormal spermatozoa genome-wide association study (GWAS) data of European from Ieu Open GWAS Project database which includes 915 cases and 209,006 control as the outcome data. Our results indicate that strict T2DM might not result in a higher risk of abnormal spermatozoa genetically in Europeans (OR: 1.017, 95% confidence interval (CI): 0.771-1.342, *p*=0.902). Our findings demonstrate that only T2DM may not explain the relatively higher risk of abnormal spermatozoa in men with it in Europeans. In subsequent studies, more comprehensive and larger samples need to be studied to reveal the relationship and potential mechanism between T2DM and abnormal spermatozoa.

## Introduction

Infertility has gradually become a global problem with a prevalence of about 10-15%, with male factors accounting for about 40% (1). The world fertility rate is still declining currently, it is predicted that the global population number will reach a peak of 9.7 billion by 2064. Subsequently, the number will be reduced to 8.8 billion by 2100 (2), and may take a severe negative effect on social development. Mammalian fertilization depends on the stability of multiple biological processes, and sperm quality is critical in sperm-oocyte penetration and activation. As many as 2% of infertile men exhibit abnormal sperm parameters (3). In addition, an insightful review has summarized that there are potential associations between male infertility and the risk of chronic disease, co-morbidities, cardiovascular disease and cancer development, so male infertility will have more possibilities as a biomarker of future health and mortality (4). Common causes and risk factors for male infertility have been postulated and/or confirmed in various studies, including smoking, alcohol consumption, drugs, obesity, past or current testicular infections, exposure to environmental toxins, testicular exposure to excessive heat, hormonal disorders, testicular trauma, and ejaculation/erectile dysfunction (5).

Spermatozoa, like other differentiated cells, have specific pathologies that can be most clearly identified by ultrastructural assessment combined with immunocytochemical and molecular techniques. This multidisciplinary approach can reveal precise structural, molecular and functional abnormalities of sperm (6). Sperm morphology is closely related to sperm motility, ability to penetrate cervical mucus, acrosome reaction process, and ability to penetrate oocyte zona pellucida. Therefore, increased sperm deformity rates will prolong natural conception and increase the risk of spontaneous miscarriage (7). Overall, it is essential to explore the risk factors of abnormal spermatozoa. Next to the traditional risk factors, emerging potential hormonal impaired biomarkers have been proposed as predictors for male infertility, such as the correlation between hyperhomocysteinemia (HHcys) and erectile dysfunction (ED) was revealed (8). So endocrine disorders or diseases that are associated with abnormal spermatozoa and male infertility deserve to be explored.

Diabetes mellitus (DM) affects an increasing number of men in their reproductive years (9). Impaired fasting glucose level (IFG) or impaired glucose tolerance (IGT) are independent parameters for the diagnosis of (DM): IFG is a state in which blood glucose levels repeatedly exceed normal blood glucose concentrations by 7 mmol/l, whereas IGT is a state in which blood glucose levels are greater than 11 mmol/l 2 hours after a 75 g oral glucose load (10). Diabetes can be classified into type 1 diabetes mellitus (T1DM), type 2 diabetes mellitus (T2DM), “other” and gestational diabetes mellitus (GDM) based on etiology and pathology (11). In the 21st century, DM has become one of the major global health issues with the development of society and improvement of quality of life (12). The number of DM patients has risen from 108 million in 1980 to 537 million (10.5%) in 2021, and it is predicted that it may increase to 643 million (11.3%) in 2030 and even to 783 million (12.2%) in 2045 (13). Studies have shown that DM has obvious negative impacts on male reproduction (10) and may affect male reproductive function on multiple levels due to its non-diabetic endocrine effects on spermatogenesis itself or by impairing erection and ejaculation (14–16). In general, DM affects male reproductive function by affecting erectile dysfunction, ejaculation, structural changes in the reproductive organs and sperm quality (17). Reactive oxygen species (ROS) overproduction, which was caused by DM, may directly or indirectly affect the entire reproductive system including the hypothalamic-pituitary-gonadal axis (HPG axis), testes tissues, epididymis and accessory glands (18).. Hyperglycemia promotes the overproduction of oxidative molecules, which, together with impaired antioxidant defenses, poses a threat to the entire body’s physiology. Not surprisingly, the reproductive organs of those patients with DM can also be injured (13, 19). Type 2 diabetes mellitus (T2DM) is the main type of DM cases diagnosed, accounting for about 85-95% (20). Numerous studies have revealed that temporary or complete infertility may occur in young adults of childbearing age with T2DM (21) T2DM can impair fertility in male animals at multiple levels, including dysregulation of endocrine control of spermatogenesis or impairing penile erection and ejaculation (22, 23). Furthermore, the low fertility rate of DM patients is well known in humans although the pathophysiological mechanisms of damage are different in T1DM and T2DM (20). There is evidence of accelerated loss of β-cell function in in younger patients with T2DM, with a prevalence estimated at 31% T2DM has increased in 10-19 years (24).

A series of studies, including clinical observations and animal studies, have focused on the effect of T2DM on sperm quality and related parameters (15, 25). However, their conclusions regarding the causal relationship between T2DM and abnormal spermatozoa were inconsistent, and the correlations between T2DM and abnormal spermatozoa risk from their studies could not fully explain the confounding of common risk factors, including socioeconomic status and unmeasured lifestyle. Mendelian randomization (MR) analysis has become a widely used tool to evaluate the causation between risk factors and outcomes using genetic variants as instrumental variables (IVs) (26). Most MR analyses were one-sample MR, performed using genetic tools, exposure, and outcomes measured in the same sample before 2011. However, MR can also be used to estimate causal effects if data on exposure and outcome are measured in different samples, which is called two-sample MR (2SMR) (27). Confounding and bias in 2SMR are limited due to the random classification of genotypes at conception (28). Therefore, in the work we used a 2SMR analysis to examine the hypothesis that T2DM is associated with the high risk of abnormal spermatozoa.

## Methods

### Study design

**Figure 1:**
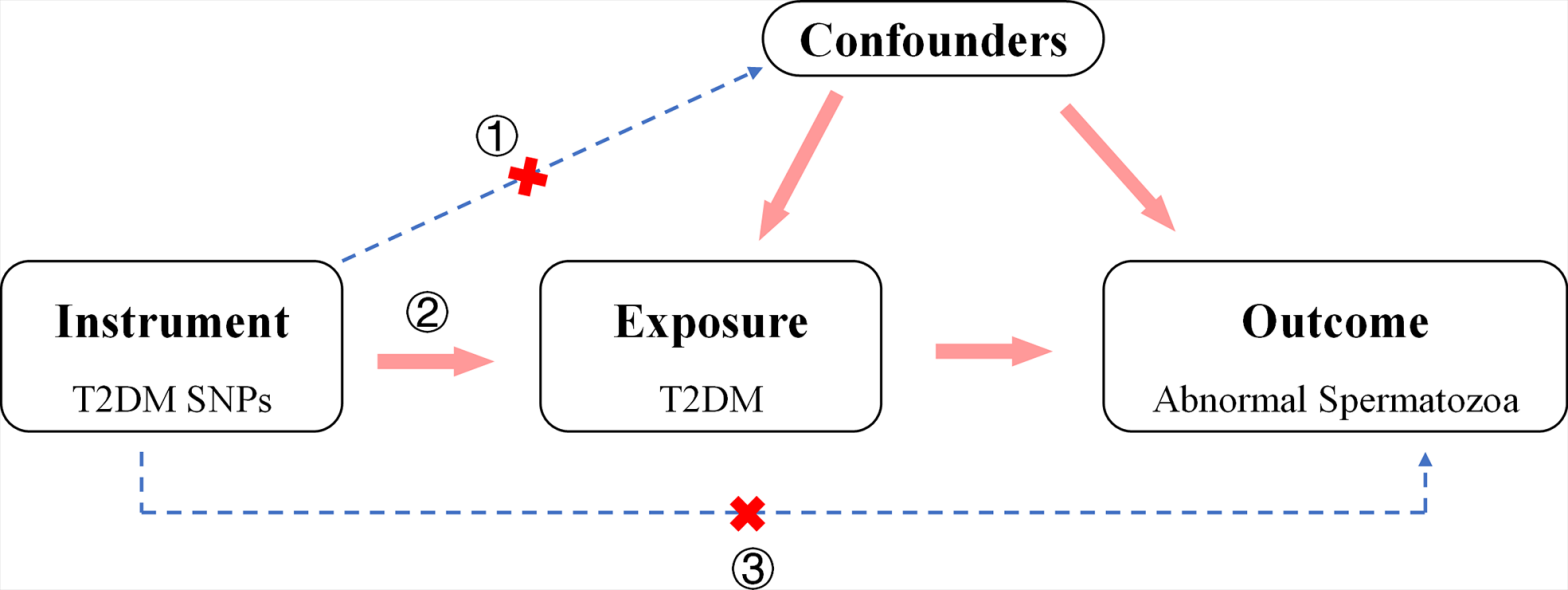
Direct Acyclic Graph of an MR analysis. The hypothesis that outcomes (abnormal spermatozoa) are caused by the exposure (T2DM) can be estimated by 2TSMR analysis. The genetic variants used in 2TSMR analysis must: a) be strongly associated with T2DM, b) be not associated with any confounder of T2DM and abnormal spermatozoa, c) be not related to the relevant outcomes obtained through other methods.

The aim of the work was to investigate the causal relationship between T2DM and the risk of abnormal spermatozoa. The genetic variants used in 2SMR analysis must: a) be strongly associated with T2DM, b) be not associated with any confounder of T2DM and abnormal spermatozoa, c) be not related to the relevant outcomes obtained through other methods (29) (**Figure 1**). Herein, we comprehensively searched for Exposure and Outcome data from Ieu Open GWAS Project database (https://gwas.mrcieu.ac.uk/) to match the most appropriate GWAS summary data. To avoid errors due to stratification effects of factors such as ancestry and population, we selected participants of European ancestry for the cohort. We preferred to use GWAS data with a larger sample size and include more SNPs and finalized the appropriate studies.

### Genetic instrumental variables for T2DM

SNPs related to T2DM in Europeans were selected in the study from a GWAS meta-analysis with GWAS-ID “ebi-a-GCST005413” that included 57,196 patients with T2DM and 12,931 health controls of European ancestry (30) (**Supplementary Table 1**). To fulfill the condition of “a) The genetic variants used in 2SMR analysis must be strongly associated with T2DM” in the study design section, those biologically and statistically plausible SNPs with a genome-wide significance threshold of *p* < 5e^-08^ were selected in our study. Furthermore, the potential weak instrumental bias and statistical power of individual SNPs were assessed by F-statistic. Weak instrument bias may ensure when F < 10, which should be excluded (31).

### Data sources of abnormal spermatozoa

For abnormal spermatozoa, Genetic association data in Europeans was acquired from the Ieu Open GWAS Project database (32), which includes a total of 915 patients with abnormal spermatozoa and 209,006 country-matched non-DM participants of European population (**Supplementary Table 1**). Herein, we extracted the overall abnormal sperm β coefficients and standard errors for each retrieved SNPs of Europeans T2DM from the GWAS summary statistics.

### 2SMR analysis methods and models

***Linkage disequilibrium assessment.*** For most MR methods, the genetic variants used in it must be unrelated with confounding factors, so linkage disequilibrium (LD) is not allowed to exist. Based on the hypothesis, the correlation LD between selected SNPs and potential confounder factors should be assessed.

***Two-sample MR analysis.*** In 2SMR study, there are five methods with a multiplicative random effects model with inverse variance weighting (IVW) as the main analysis method and four other robust methods: MR-Egger, Weighted median, Weighted mode and Sample mode (33). Separately, the IVW method is the most widely used and accepted MR method because it is the most effective method in the presence of a valid IV and heterogeneity also can be taken into account when analyzing causality (32). The MR-Egger method provides a gradually consistent causal effects measurement that adjusts for horizontal pleiotropy by pooling a single SNP-specific Wald ratio through adaptive Egger regression (32). The weighted median method yields a gradually consistent causal effect estimate by using the weighted median of Wald ratios, provided that at least 50% of the variants meet a valid IV for the exclusion restrictions. The WMO method groups SNPs based on their similarity based on their estimation of individual proportions, then calculates the counter variance weighted number of SNPs in each group, and finally derives a causal estimate based on the group of SNPs with the largest weighted number (34). The Simple medians provide consistent estimates of causal effects if at least 50% of the IVs are valid (35).

***Leave-one-out sensitivity and Heterogeneity analysis.*** The leave-one-out sensitivity analysis can be used to come to an assessment of the influence of causal estimates by individual SNPs. Heterogeneity was assessed by Cochran’s Q statistic and associated *p*-values as an indicator to evaluate whether the causal relationships were consistent of all the SNPs, where smaller heterogeneity suggests more reliable MR estimates.

***MR Pleiotropy Residual Sum and Outlier (MR-PRESSO) analysis.*** In 2SMR study, the MR-PRESSO method was used to analyze the pleiotropy of IVs and correct abnormal results caused by outliers. In detail, It consists of 3 steps: MR-PRESSO global test was used to identify heterogeneity and outliers, subsequently using MR-PRESSO outlier test to correct for pleiotropy by excluding outliers, and finally analyzing the difference in causality before and after outlier removal with MR-PRESSO distortion test (36).

### Statistical analysis

MR is based on the principle of random distribution of genetic genes. When the frequency of SNPs is highly consistent with the change of exposure variables, it can be preliminarily considered that the SNP is related to the exposure variable. In the 2SMR study, those SNPs with LD-R2 < 0.001 were retrieved by linkage disequilibrium assessment. The casual relationship between T2DM and the risk of abnormal spermatozoa was estimated using five methods of 2SMR with IVW as the main analysis method. Leave-one-out sensitivity analysis, heterogeneity analysis and MR-PRESSO analysis were used to analyze the reliability of the pleiotropy of IVs and correct abnormal results caused by outliers.

## Results

### Selected SNPs and IVs validation

The identification and information on genetic variants associated with T2DM in this study were derived from a GWAS meta-analysis conducted in 2018 by Bonàs-Guarch S et al. (30), 17 single nucleotide polymorphisms (SNPs) among participants of European ancestry were estimated to be correlated to T2DM at the significant difference level (*p* < 5×10^-8^). Among them, 9 independent SNPs with F-statistics > 10 and all of them surpassed the limited value (r^2^ < 0.001) in LD analysis, we then retrieved the genes to which each SNPs belonged and summarized their detailed information in a table (**Table 1**).

**Table 1:** T2DM SNPs used to construct the instrument variable in Europeans.

Chr	Position		SNP	Gene	EA	OA	EAF	Beta	SE	P value
1	40035928		rs3768321	PABPC4	T	G	0.1965	0.1121	0.0195	9.02E-09
2	227121918		rs2943656	IRS1	G	A	0.6344	0.1034	0.0164	2.74E-10
3	185519107		rs71320321	IGF2BP2	A	G	0.3184	0.136	0.0168	5.05E-16
5	55856375		rs3843467	C5orf67	T	G	0.2032	0.1223	0.0192	1.90E-10
6	20675792		rs35261542	CDKAL1	A	C	0.2765	0.1331	0.0173	1.66E-14
6	32428115		rs9268835	DRB3	A	G	0.2914	0.1276	0.0189	1.37E-11
9	22136440		rs12555274	CDKN2A	C	G	0.2666	0.1256	0.0197	1.67E-10
10	12307894		rs11257655	CDC123	T	C	0.2097	0.1204	0.0192	3.74E-10
10	114754071		rs34872471	TCF7L2	C	T	0.701	0.3529	0.0171	1.15E-94

Chr, chromosome; SNP, single nucleotide polymorphism; EA, Effect Allele; OA, Other Allele; EAF, effect allele frequency; SE, standard error.

### MR assessing T2DM effects on abnormal spermatozoa

**Figure 2:**
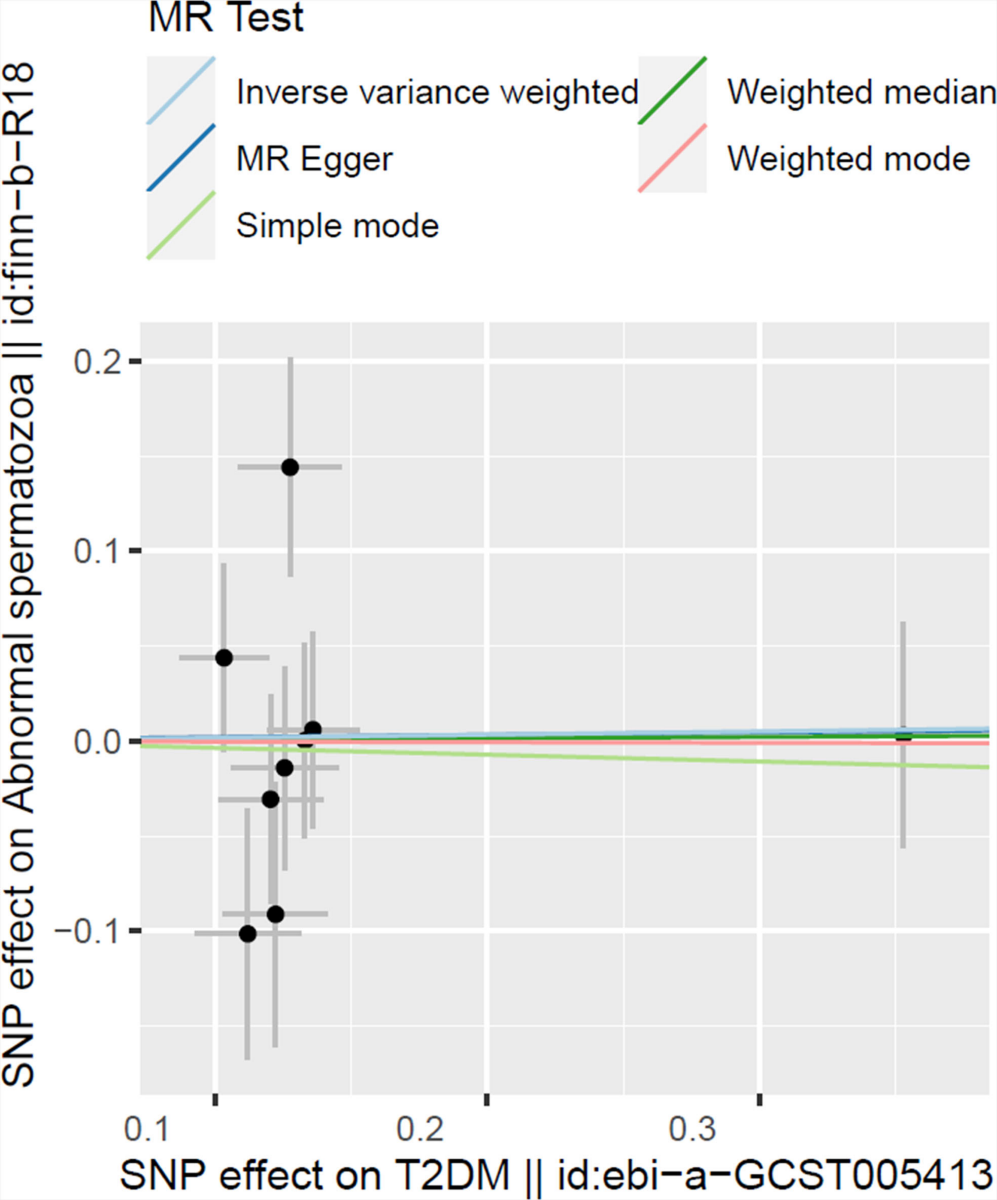
Scatter plot illustrating the distribution of individual ratio estimates of T2DM with abnormal spermatozoa as the outcome. Trend lines derived from five different 2SMR methods are also included in each scatter plot to indicate cause and effect.

To test the T2DM effects on the risk of abnormal spermatozoa, we have adopted five 2SMR methods in this work and the results are organized in **Table 2** and **Figure 2**. Among them, the IVW was used in our work as the main method to estimate the causal effect of T2DM on the risk of abnormal spermatozoa. However, none of the results in our work found a significant causal relationship between T2DM and the risk of abnormal spermatozoa in European population (IVW method, OR:1.017, 95% CI: 0.771-1.342, *p*=0.902).

**Table 2:** Associations between genetically predicted T2DM and risk of abnormal spermatozoa.

Methods	OR	95%CI of OR	P value
Inverse-variance Weighted	1.017	0.771-1.342	0.902
MR-Egger	1.012	0.509-2.015	0.972
Simple mode	0.965	0.608-1.530	0.881
Weighted median	1.007	0.752-1.349	0.961
Weighted mode	0.997	0.751-1.324	0.984

OR, odds ratio; CI, confidence interval.

### Assessment of MR assumptions

**Figure 3:**
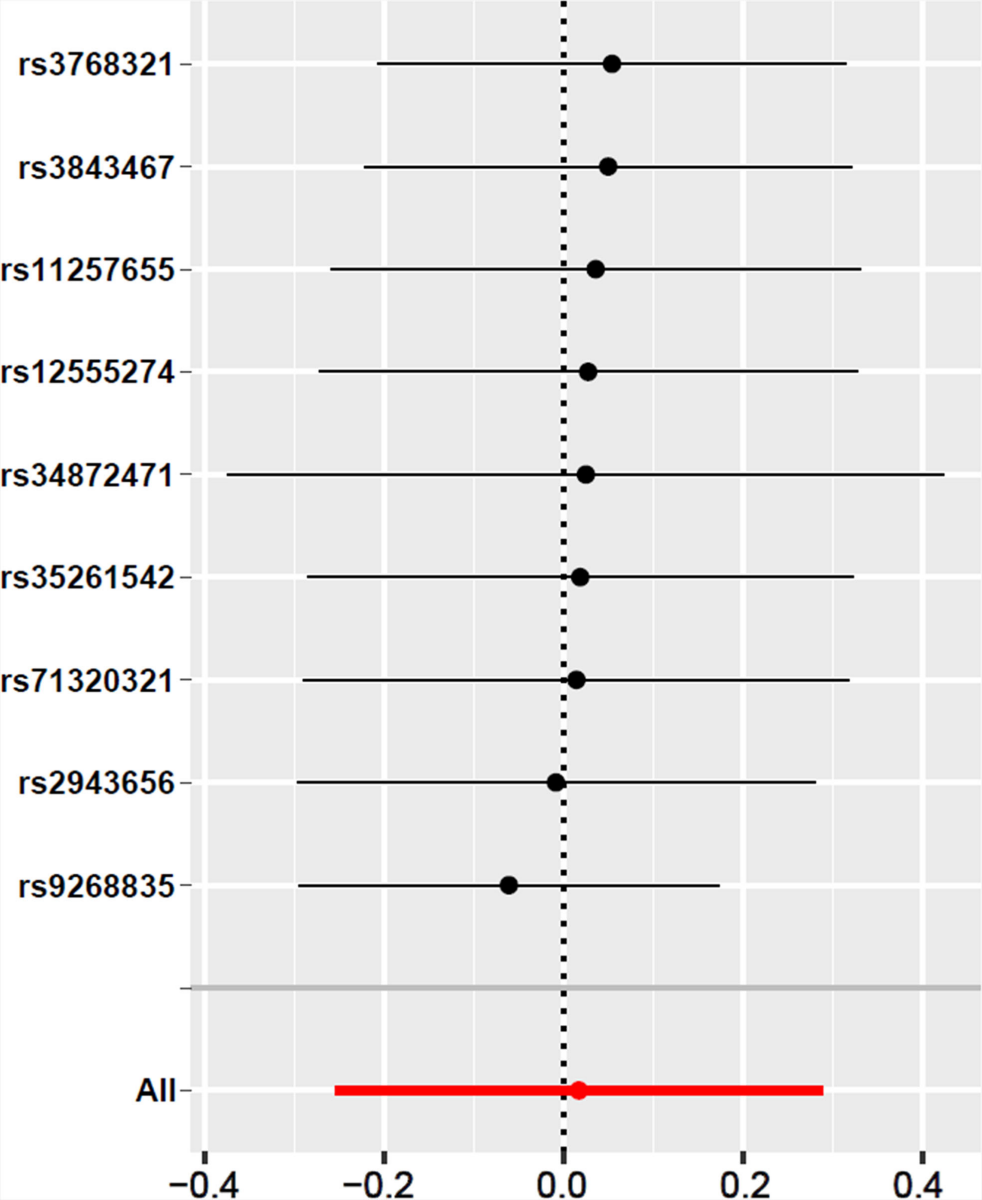
Leave-one-out sensitivity analysis for T2DM on abnormal spermatozoa. The given dark dots indicate effect measures from IVW-MR analysis excluding specific SNPs. Red lines indicate pooled analysis including all SNPs by the IVW-MR method (plotted for comparison).

In the work, we have selected those SNPs at the genome-wide significance level of *p* < 5 × 10^–8^ to comply with our first condition. Leave-one-out analysis also showed that there has no evidence of a significant effect of individual SNPs on the overall effect of T2DM on abnormal spermatozoa (**Figure 3**). The heterogeneity analysis results showed that there has no statistically significant heterogeneity in all of five 2SMR analysis (*p* > 0.05) (**Table 3**). In addition, we haven’t found significant horizontal pleiotropy from the results of pleiotropy analysis (*p* > 0.05) (**Table 4**). These results suggest that the causal estimate between T2DM and the risk of abnormal spermatozoa didn’t receive confounding factors. Furthermore, the results obtained from the MR-PRESSO analysis confirmed that there have no significant horizontal pleiotropy and outliers exist in the study (*p* > 0.05) (**Table 5**), which is consistent with the results above.

**Table 3:** Heterogeneity statistics of two-sample Mendelian randomization analysis.

Method	Q value	Degrees of freedom	*p-*value
Inverse-variance weights	11.491	7	0.119
MR-egger	11.491	8	0.175

**Table 4:** Pleiotropy statistics of two-sample Mendelian randomization analysis.

Method	Egger regression intercept	Standard error	Directionality p-value
MR-egger	0.000798	0.0555	0.9889

**Table 5:** Pleiotropy and outliers statistics of MR-PRESSO analysis.

	Exposure	MR Analysis	Casual Estimate	Standard Deviation	T-statistics	*p*-value
Main MR	T2DM	Raw	0.017	0.139	0.123	0.905
	T2DM	Outlier-corrected	NA	NA	NA	NA
RSSobs of Global Test in MR-PRESSO results			12.900			
P value of Global Test in MR-PRESSO results			0.291			

NA, Not applicable.

## Discussion

Unlike the risk factors for abnormal spermatozoa, such as smoking, alcohol consumption and inflammation, we aim to reveal the association between the endocrine disease T2DM and the risk of abnormal spermatozoa. As far as we know, our work is the first 2SMR study for the casual correlation between T2DM and the risk of abnormal spermatozoa using large-sample cohorts of European ancestry. Through MR analysis, we found that there has no causal relationship between T2Dm and the risk of abnormal spermatozoa in European population.

A insightful review indicated that DM can permanently damage various organs and lead to dysfunction or failure and numerous studies in both humans and animals have pointed out the negative effect of DM on male reproduction function (37). Although they summarize the effects including T1DM and T2DM, our findings may still contradict this. T2DM, accounting for approximately 85-95% of diagnosed DM cases, is the main type of DM (20), and has negative effects on multiple physiological systems, including the reproductive system. Currently, the incidence of T2DM is increasing rapidly among adolescents, especially men, which will lead to a significant increase in their prevalence of reproductive dysfunction (38).. In a retrospective analysis study, they found a 51% prevalence of subfertility among patients with T2DM (39). In another study, researchers surveyed more than 500 male partners of infertile couples and found that about 1.2% of infertile men had T2DM (40).. A new study showed that the prevalence of infertility in men with T2DM has achieved 35.1% and it was significantly higher compared with normal participants (41). Lots of studies show that DM often affects the fertilization process in males by inducing reactive oxygen species (ROS), which have negative impacts on the development of sperm (42–45).

An early study evaluated sperm quality in patients with T1DM and T2DM, whose semen analysis showed qualitative alterations, the main effect is on the dynamics, especially on the progressive kinematics (40).. In addition, a study in 2018 also found reduced sperm count and viability in men with T2DM (46). Another clinical study found that DM had significant negative effects on the quality of sperm parameters, including motility and concentration, as well as increased morphological abnormalities, and that sperm DNA/chromatin levels were substantially altered in DM patients, affecting sperm maturation process (41). In contrast to the morphological and motility findings, researchers noted a decrease in semen volume in DM men compared to other patients, while no significant changes in other semen parameters were observed (14), this may be consistent with our conclusion. Studies in animal models of T2DM have revealed that it may lead to dysregulated spermatogenesis, disturbances in endocrine control, or impairment of erectile function and ejaculation disorders, thereby impairing male fertility (22, 23).. In addition, the treatment of rats with T2DM restored steroidogenesis in their testes, resulting in improved spermatogenesis (47). Other studies also showed that treatment of T2DM increased sperm survival after 24 hours of storage in pigs and improved the quality of frozen sperm in dogs (48). However, inconsistent effects of T2DM treatment on sperm count, concentration, morphology, viability and survival were found in *in vivo* studies from a variety of animal models, including rats, mice/rats, rabbits and fish (46).

We suppose that the inconsistent findings of those studies may be caused by the effects of confounding factors, hence the major advantage of MR which can remove the effects of confounding factors is realized (49). Therefore, we designed a 2SMR study to reveal the causal association between T2DM and the risk of abnormal spermatozoa. In the present 2SMR study, we found that there has no causal association between T2DM and the risk of abnormal spermatozoa in European population. Nevertheless, our work contains several advantages. The study’s IVs and abnormal spermatozoa data are obtained from the most suitable GWAS data in Europeans, which allows for better representation of exposure and outcomes. Furthermore, the study fulfilled 3 assumptions. In detail, we retrieved 9 SNPs that were strongly associated with T2DM (*p* < 5e^-08^) and exclude instrument bias (F < 10) from GWAS, which fulfilled the first assumption. To ensure that the genetic variants used in the study were not related with exposure or outcome confounding factors–the second assumption, the linkage disequilibrium (LD) analysis was used and all of 9 SNPs surpassed the limited value (R^2^ < 0.001), but the reverse result cannot be excluded due to the presence of unmeasured confounding factors. Finally, the third assumption was assessed, Leave-one-out sensitivity analysis, Heterogeneity analysis, and MR-PRESSO analysis were used in the study and didn’t find horizontal pleiotropy.

However, there are still some limitations in our study. First, we only matched data for the European population, which is hardly representative of the total population. Second, the number of cases of abnormal spermatozoa may not be enough, thus there may be bias in the study. Third, we were unable to correlate the different classifications of abnormal spermatozoa due to a lack of detailed data. But the limitations in our study also suggest a worthwhile direction for future research so that the causal relationship between T2DM and abnormal sperm can be better revealed.

## Conclusion

In conclusion, through MR analysis using data summaries from large sample GWAS analysis, our results suggest that there has no causal association between T2DM and the risk of abnormal spermatozoa in European population. More comprehensive and larger size GWAS data need to be established to explore the causal association between T2DM and different types of abnormal spermatozoa.

## Data availability statement

The original contributions presented in the study are included in the article/**Supplementary Material**, further inquiries can be directed to the corresponding author.

## Author contributions

Conceptualization: JG and YZ; data curation: MR analysis: MD; funding acquisition: JG and YZ; software and visualization: WG and MC; writing—original draft: MD; writing—review and editing: YZ and GG. MD has verified the underlying data. All the authors approved the final version of the manuscript.

## Funding

National Natural Science Foundation of China (YZ, Grant No. 82001496), project of Chengdu Science and Technology Bureau, (YZ, Grant No. 2021-YF05-02110-SN), China Postdoctoral Science Foundation (YZ, Grant No. 2020M680149, 2020T130087ZX).

## Acknowledgments

Data in the European population on T2DM and abnormal spermatozoa are available through the UK Biobank and data analysis is available through the GWAS database. The authors thank these researchers for their selfless sharing.

## Conflict of interest

The authors declare that the research was conducted in the absence of any commercial or financial relationships that could be construed as a potential conflict of interest.

The reviewer FQ declared a shared affiliation with the authors to the handling editor at the time of review.

## Publisher’s note

All claims expressed in this article are solely those of the authors and do not necessarily represent those of their affiliated organizations, or those of the publisher, the editors and the reviewers. Any product that may be evaluated in this article, or claim that may be made by its manufacturer, is not guaranteed or endorsed by the publisher.
