# Supplementary material for: Type 2 diabetes mellitus and the risk of abnormal spermatozoa: A Mendelian randomization study
Source: Front Endocrinol (Lausanne). 2022 Nov 2;13:1035338. doi: 10.3389/fendo.2022.1035338 (PMC9666365; doi:10.3389/fendo.2022.1035338)
Supplement: Supplementary file 1 [file Table_1.docx]

Supplementary Material

# Supplementary Table

**Supplementary Table 1. Details of studies included in Mendelian randomization analyses**

| Trait | Number of cases | Number of controls | Sample size | Number of SNPs | Year | Population |
| --- | --- | --- | --- | --- | --- | --- |
| T2DM | 12,931 | 57,196 | 70,127 | 14,277,791 | 2018 | European |
| Abnormal Spermatozoa | 915 | 209,006 | 209,921 | 16,380,442 | 2021 | European |
